# Vitamin D and bone — How does vitamin D regulate bone formation and resorption? —

**Published:** 2004-09-01

**Authors:** Tatsuo Suda

**Affiliations:** Division of Pathophysiology, Research Center for Genomic Medicine Saitama Medical School, 1397-1 Yamane, Hidaka, Saitama 350-1241

**Keywords:** Vitamin D, 1***α***,25-dihydroxyvitamin D_3_, bone formation and resorption, osteoblasts, osteoclasts, receptor activator of NF***κ*** B ligand (RANKL)

## Abstract

Vitamin D was discovered as an anti-rachitic agent, but even at present, there is no direct evidence to support the concept that vitamin D directly stimulates osteoblastic bone formation and mineralization. It appears to be paradoxical, but vitamin D functions in the process of osteoclastic bone resorption. Osteoclasts, the only cells responsible for bone resorption, develop from hematopoietic cells of the monocyte-macrophage lineage. In 1992, we hypothesized that a membrane-bound factor, designated as “osteoclast differentiation factor (ODF)”, is expressed on the plasma membrane of osteoblasts/stromal cells in response to osteotropic factors including the active form of vitamin D_3_, 1***α***,25-dihydroxyvitamin D_3_ [1***α***,25(OH)_2_D_3_]. Recently, four research groups including ours independently identified three key molecules (RANKL, RANK, and OPG) responsible for osteoclastogenesis. A long-sought-after ligand, ODF, was identical to RANKL. RANKL was a member of the membrane-associated TNF ligand family, which induced differentiation of spleen cells (osteoclast progenitors) into osteoclasts in the presence of M-CSF. RANK, a member of the TNF receptor family, was a signaling receptor essential for the RANKL-mediated osteoclastogenesis. OPG, a secreted member of the TNF receptor family, was a decoy receptor for RANKL. The discovery of RANKL, RANK and OPG opens a new era in the study of bone biology and the therapy of several metabolic bone diseases such as osteoporosis, rheumatoid arthritis, and periodontal diseases.

## Introduction

Bone is a dynamic tissue that is formed and remodeled by continuously occurring bone formation and resorption. An imbalance between bone formation and resorption causes several metabolic bone diseases such as osteoporosis and osteopetrosis. Bone-forming osteoblasts derive from undifferentiated mesenchymal cells, whereas bone-resorbing osteoclasts develop from hemopoietic cells of the monocyte-macrophage lineage. The hemopoietic osteoclast precursor cells differentiate into osteoclasts only at bone-resorbing sites under the control of several osteotropic hormones and cytokines.1)

**Fig. 1:**
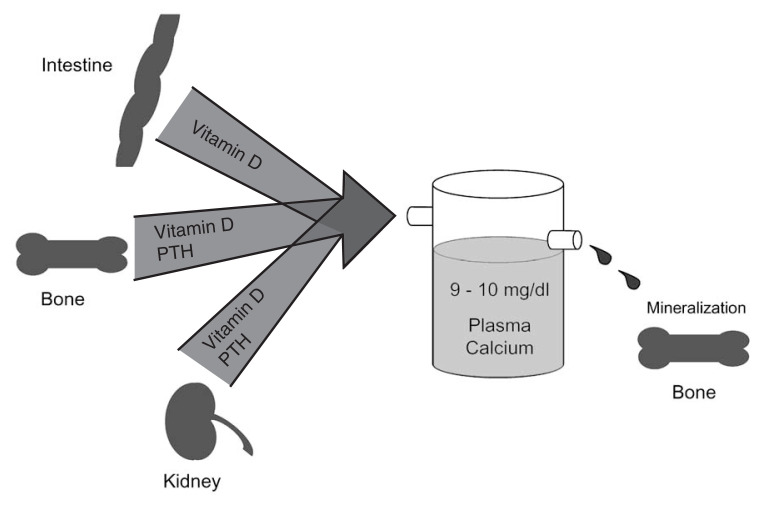
Diagrammatic representation of the classical actions of vitamin D. While vitamin D, through its active metabolite 1***α***,25(OH)_2_D_3_, is the only substance to stimulate intestinal absorption of calcium, vitamin D and parathyroid hormone (PTH) working in concert are necessary to mobilize calcium from bone and conserve calcium from urine.

It is well recognized that, in healthy animals and humans, serum calcium levels are tightly regulated and maintained at 9 to 10 mg/dl.2) Intestine, bone, and kidney are the three major organs involved in this calcium homeostasis. Vitamin D plays a major role in regulating serum calcium homeostasis in concert with parathyroid hormone (PTH). Of great importance is the fact that PTH is required for calcium mobilization from bone and for renal reabsorption of calcium, but is not directly required for intestinal calcium absorption 2) (Fig. 1). The total amount of calcium present in the body is estimated to be approximately 1,000 gram, and 99% of them are stored as an available form in bone. Bone calcium is mobilized by osteoclasts into blood from calcified bone. Thus, it is considered that bone is a storehouse of calcium in the body.2) Vitamin D was discovered as an antirachitic agent. A deficiency of vitamin D results in rickets in the young or osteomalacia in the adult. Although it had been believed that vitamin D plays an important role in the process of mineralization *per se*, there is no direct evidence to support this concept even at present.2)

I have been involved in the study on vitamin D biochemistry and bone biology for over 40 years. While I was studying abroad at the University of Wisconsin, I had a chance to isolate and identify the active form of vitamin D_3_ [1***α***,25(OH)_2_D_3_] under the guidance of Professor Hector F. DeLuca. In this article, I review the outline of vitamin D metabolism and the role of 1***α***,25(OH)_2_D_3_ in bone formation and resorption.

## Metabolism of Vitamin D—Discovery of the active form of vitamin D, 1*α*,25(OH)_2_D_3_

Vitamin D was originally identified as a fat-soluble vitamin, the physiological role of which is to regulate calcium metabolism.2) Vitamin D consists of two forms; vitamin D_2_ (ergocalciferol, a plant vitamin D) and vitamin D_3_ (cholecalciferol, an animal vitamin D). The biopotency of vitamin D_2_ is equal to that of vitamin D_3_ in humans (40 IU/μg).2) A deficiency of vitamin D results in rickets in the young and osteomalacia in the adult. Administration of vitamin D into rachitic animals and humans cures impaired bone formation and mineralization. From these results, it was postulated that vitamin D stimulates osteoblastic bone formation and mineralization *per se*, but there is no direct evidence to support this concept even at present.2) DeLuca has reported that vitamin D does not function directly in either bone growth or mineralization, when plasma calcium and phosphorus levels are maintained in a normal range.2)

**Fig. 2:**
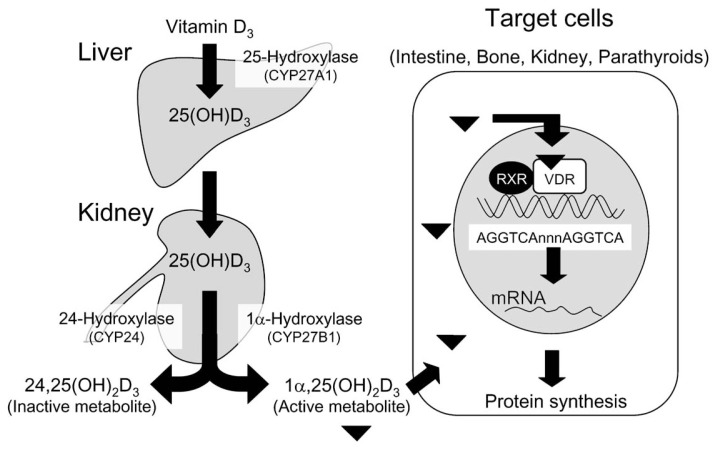
Metabolism of vitamin D_3_ and mode of action of its active metabolite 1***α***,25(OH)_2_D_3_ in the target cells. Vitamin D_3_ is converted to its active metabolite 1***α***,25(OH)_2_D_3_ by two successive hydroxylations first in the liver, then in the kidney. 1***α***,25(OH)_2_D_3_ secreted from the kidney is transported to the target cells and induces its actions by a genomic mechanism.

It is well known that 7-dehydrocholesterol (provitamin D_3_) is converted in the skin to vitamin D_3_ by irradiation with ultraviolet light. Vitamin D_3_ is then metabolized in the liver 3) by 25-hydroxylase (CYP27A1)4),5) into 25-hydroxyvitamin D_3_ [25(OH)D_3_] 6),7)). This compound must be subsequently metabolized in the kidney8) into either 1***α***,25-dihydroxyvitamin D_3_ [1***α***,25(OH)_2_D_3_]9),10) by 1***α***-hydroxylase (CYP27B1)11)–14) or 24,25-dihydroxyvitamin D_3_ [24,25(OH)_2_D_3_]15) by 24-hydroxylase (CYP24).16) Of these two dihydroxymetabolites, 1***α***,25(OH)_2_D_3_ has been recognized to be the final active form of vitamin D_3_ 17) (Fig. 2). Renal production of 1***α***,25(OH)_2_D_3_ is strongly stimulated by PTH and inhibited by 1***α***,25(OH)_2_D_3_, whereas that of 24,25(OH)_2_D_3_ is strongly inhibited by PTH and induced by 1***α***,25(OH)_2_D_3_.17)–19) It is well known that this type of metabolic regulation of vitamin D by PTH and 1***α***,25(OH)_2_D_3_ is particularly important physiologically to maintain plasma calcium homeostasis.17)–19) 1***α***,25(OH)_2_D_3_ is now recognized to be a steroid hormone generated in the kidney to control calcium metabolism. 17)

1***α***,25(OH)_2_D_3_ secreted into blood is transported to the target organs of vitamin D including intestine, bone and kidney, then easily penetrates plasma membranes of the target cells to bind nuclear 1***α***,25(OH)_2_D_3_ receptor (VDR).20),21) To date, only single receptor has been identified.21) The interaction of 1***α***,25(OH)_2_D_3_ with VDR requires a retinoid receptor (RXR).21) The vitamin D responsive element (VDRE) has been identified in the 5′ upstream region of the target genes of 1***α***,25(OH)_2_D_3_ including osteocalcin, calbindin D_28K_, 24-hydroxylase (CYP24), and PTH genes.20)–23) The DNA sequence of VDRE is a direct repeat structure with a 3 nucleotide base spacer20)–23) (Fig. 2).

Yoshizawa *et al*.24) first succeeded in generating mice deficient in VDR by gene targeting. They showed that in VDR null mutant (KO) mice, no appreciable defects were observed in development and growth before weaning, irrespective of the reduced expression of vitamin D target genes. After weaning, however, mutant mice failed to thrive, and alopecia, hypocalcemia and infertility resulted.24) Both bone formation and mineralization were severely impaired as a typical feature of type II vitamin D-dependent rickets. Most of the VDR KO mice died within 15 to 25 weeks after birth due to severe hypocalcemia. Unexpectedly, when these VDR KO mice were fed a rescue diet containing high calcium, they developed normally even at weeks 50, but severe alopecia remained.24) Bone formation and mineralization in the VDR KO mice maintained on the high calcium diet were completely reestablished by the high calcium diet feeding. From these results, it was concluded that the stimulating effect of 1***α***,25(OH)_2_D_3_ on bone formation and mineralization is indirect, occurring through the stimulation of intestinal absorption of calcium by vitamin D. Although a non-genomic mechanism of the action of 1***α***,25(OH)_2_D_3_ has also been postulated,25) firm evidence to support this concept is lacking. Thus, it is concluded that vitamin D functions preferentially via its single receptor VDR by a genomic mechanism2),21) (Fig. 2).

Very recently, Tanaka and Seino26) examined direct action of vitamin D on bone formation and mineralization by transplanting bone isolated from VDR KO mice to wild type (WT) mice. The VDR KO bone transplanted to the WT mice showed excessive bone formation and mineralization in normocalcemic conditions. This suggests that vitamin D negatively regulates bone formation and mineralization. Thus, the stimulating effect of l***α***,25(OH)_2_D_3_ on bone formation and mineralization appears to be due to preferentially the stimulation of intestinal absorption of calcium by vitamin D. The vitamin rather directly regulates osteoblastic bone formation by inhibiting excessive bone formation and mineralization.

## Vitamin D mobilizes calcium from calcified bone

It appears to be paradoxical, but vitamin D functions in the process of calcium mobilization from calcified bone, making calcium available to the extracellular fluid upon demand by the calcium homeostatic system. Carlsson27) reported for the first time this important observation. He showed that, when hypocalcemic rats maintained on a vitamin D-deficient, low calcium diet were orally given 100 IU (2.5 μg) of vitamin D_3_, their serum calcium was increased from 5 to 8 mg/dl 3 days after vitamin D_3_ administration. Parathyroidectomy 2 hours prior to vitamin D_3_ administration abolished the increase in serum calcium levels. Since the diet did not contain any appreciable amounts of calcium, he concluded that vitamin D stimulates mineral mobilization from calcified bone to blood in concert with PTH.27)

The metabolite of vitamin D_3_ responsible for bone mineral mobilization was 1***α***,25(OH)_2_D_3_. Using an *in vitro* organ culture system, Raisz *et al*.28) reported that both 1***α***,25(OH)_2_D_3_ and 25(OH)D_3_ increase the release of ^45^Ca from prelabeled bone into the culture medium, but 1***α***,25(OH)_2_D_3_ is 80 times more potent than 25(OH)D_3_. From these results, they concluded that the metabolite of vitamin D_3_ which stimulates bone mineral mobilization is indeed 1***α***,25(OH)_2_D_3_. Abe *et al*.29) discovered the cell differentiation-inducing activity of 1***α***,25(OH)_2_D_3_ using mouse and human myeloid leukemia cells. HL-60 is a human promyelocytic leukemia cell line established from a leukemic patient, and this cell line can be induced to differentiate into granulocytes by retinoic acid and monocytes-macrophages by 1***α***,25(OH)_2_D_3_. 1***α***,25(OH)_2_D_3_ was a potent and selective inducer of differentiation of HL-60 cells into macrophages.30) Furthermore, 1***α***,25(OH)_2_D_3_ directly induced fusion of alveolar macrophages at a very high rate.31) Approximately 80% of the macrophages fused to form multinucleated giant cells by stimulating the differentiation and fusion of macrophages. However, the multinucleated giant cells formed from alveolar macrophages in response to 1***α***,25(OH)_2_D_3_ did not satisfy the criteria of osteoclasts.

## Establishment of a co-culture system for generating osteoclasts *in vitro*

In 1981, Rodan and Martin32) proposed that osteoblasts or bone marrow stromal cells may intervene in the process of osteoclastic bone resorption. Their argument for such a mechanism was based on the observations that first, bone-resorbing hormones and cytokines have their receptors in osteoblastic cells but not in osteoclasts, and second, the relative binding potencies of these bone-resorbing hormones and cytokines to their respective receptors in osteoblasts resemble those in inducing bone resorption. 32) The same conclusion was reached independently by Chambers,33) who proposed that a factor called osteoclast activating factor (OAF) is produced by osteoblastic cells in response to bone-resorbing hormones and cytokines, then stimulates osteoclast activation.33)

**Fig. 3:**
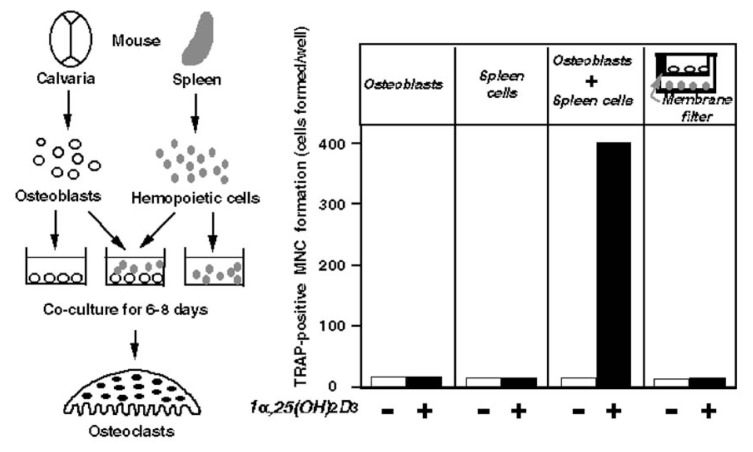
A mouse co-culture system to recruit osteoclasts. Primary osteoblasts from the calvaria and/or hemopoietic cells from the spleen were cultured for 6–8 days in the presence or absence of 10^−8^ M 1***α***,25(OH)_2_D_3_. Tartrate-resistant acid phosphatase (TRAP)-positive multinucleated cells (MNC), which were identified as osteoclasts, were formed only when osteoblasts and spleen cells were co-cultured in the presence of 1***α***,25(OH)_2_D_3_.

To examine the possible involvement of osteoblastic cells in osteoclast formation, we established an efficient mouse co-culture system to recruit osteoclasts34) based on the concept proposed by Rodan and Martin.32) In this co-culture system, primary osteoblastic cells were isolated from mouse calvaria, and spleen cells isolated from the splenic tissue were used as osteoclast progenitors34) (Fig. 3). When osteoblastic cells alone or spleen cells alone were cultured, no osteoclasts were formed even in the presence of 1***α***,25(OH)_2_D_3_. Multinucleated osteoclasts were formed only when spleen cells and osteoblastic cells were co-cultured in the presence of 1***α***,25(OH)_2_D_3_ 34) (Fig. 3). Cell-to-cell contact between spleen cells and osteoblastic cells appeared important for osteoclast formation, since no osteoclasts were formed when they were co-cultured but separated by a membrane filter. No osteoclasts were formed in the absence of 1***α***,25(OH)_2_D_3_ even in the co-culture. Taken together, we hypothesized that the direct contact of spleen cells and osteoblastic cells was essential for osteoclast differentiation. Spleen cells represent osteoclast progenitors, in other words “**seeds**”, and osteoblastic cells represent the supporting cells to provide a suitable microenvironment for osteoclast formation in bone, in other words “**farm**”.

## A hypothetical factor: osteoclast differentiation factor (ODF)

**Fig. 4:**
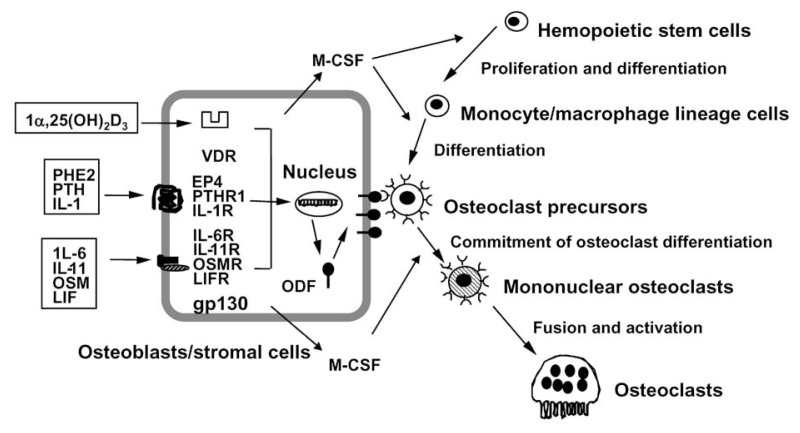
A hypothesis for the possible mechanisms of osteoclast development. Osteotropic factors including 1***α***,25(OH)_2_D_3_, PTH and IL-11 stimulate osteoclast formation in co-cultures of osteoblasts/stromal cells and hemopoietic cells. Osteoblasts/stromal cells express a membrane-associated factor called ODF in response to several osteotropic factors. Osteoclast progenitors of the monocyte-macrophage lineage recognize ODF in osteoblasts/stromal cells through cell-to-cell interaction, then differentiate into osteoclasts in the presence of M-CSF.

In 1992, we proposed a working hypothesis for the mechanism of osteoclastogenesis based on the extensive studies using the co-culture system. Various bone-resorbing hormones and cytokines including 1***α***,25(OH)_2_D_3_, PTH and interleukin (IL)-11 appeared to act commonly on osteoblastic cells, but not on hemopoietic osteoclast precursors in co-cultures of osteoblastic cells and spleen cells1) (Fig. 4). These bone-resorbing factors were classified into three categories in terms of their signal transduction pathways: VDR-mediated signals [1***α***,25(OH)_2_D_3_], protein kinase A-mediated signals [PTH, prostaglandin E_2_ (PGE_2_) and IL-1], and gp130-mediated signals [IL-6, IL-11, oncostatin M (OSM), and leukemia inhibitory factor (LIF)].1) These three diverse signals appeared to stimulate osteoclast formation independently, since VDR-KO mice and gp130-KO mice possessed osteoclasts in bone tissues *in vivo*. We proposed that a membrane-bound factor(s), which is commonly induced on osteoblastic cells in response to these bone-resorbing factors, mediates an essential signal to osteoclast progenitors for their differentiation into mature osteoclasts (Fig. 4). We named the factor “osteoclast differentiation factor” (ODF).35) ODF appeared to be identical to “stromal cell-derived osteoclast formation activity (SOFA)” proposed by Chambers *et al*.36) Osteoclast progenitors having ODF receptor recognize ODF by cell-to-cell contact and differentiate into osteoclasts. Macrophage colony-stimulating factor (M-CSF) produced by osteoblastic cells is also indispensable for both proliferation and differentiation of osteoclast progenitors.37) Yoshida *et al*.38) demonstrated that osteopetrotic (*op*/*op*) mutant mice with a defect in the development of osteoclasts have a loss of function mutation in the coding region of M-CSF gene. Thus, osteoblastic cells are important for osteoclast recruitment in two different ways: one is the production of M-CSF, and the other is the production of a membrane-bound factor such as ODF commonly induced by several bone-resorbing factors1),35) (Fig. 4).

## Discovery and Identification of OPG/OCIF

**Fig. 5:**
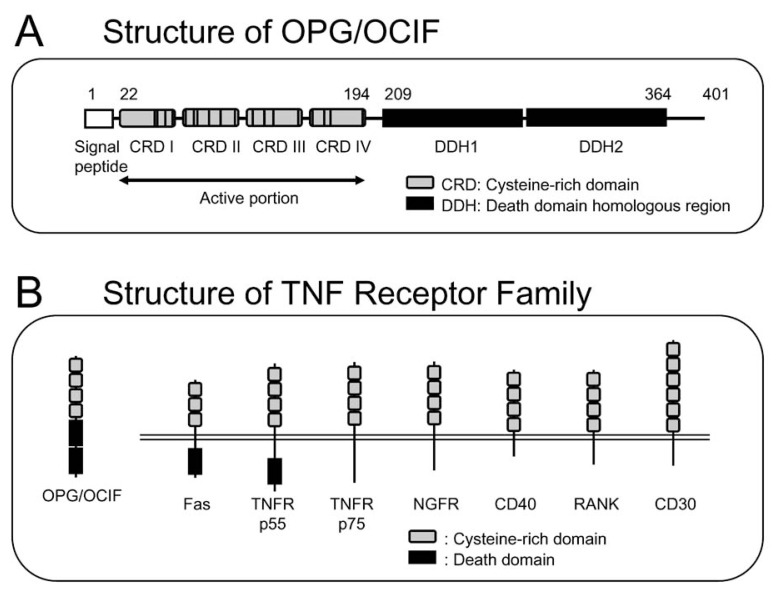
Structure and functional domains of human OPG/OCIF. (A) Diagrammatic representation of the structure of OPG/OCIF. (B) Comparison of the structure of OPG/OCIF and other TNF receptor family members.

In 1997, Simonet *et al*.39) cloned a new member of the tumor necrosis factor (TNF) receptor family, termed osteoprotegerin (OPG). Interestingly, OPG lacked a transmembrane domain and represented a secreted TNF receptor (Fig. 5). Hepatic expression of OPG in transgenic mice resulted in osteopetrosis.39) Tsuda *et al*.40) independently isolated a novel protein termed osteoclastogenesis inhibitory factor (OCIF) from conditioned medium of human fibroblasts (IMR90) by using bone marrow cell cultures treated with 1***α***,25(OH)_2_D_3_ as an assay system for *in vitro* osteoclast formation. The cDNA sequence of OCIF was identical to that of OPG.39),40)

OPG/OCIF consisting of 401 amino acid residues contained four cysteine-rich domains and two death domain homologous regions (Fig. 5).39),41) The death domain homologous regions share structural features with “death domains” of TNF receptor p55, Fas and TRAIL receptors, which mediate apoptotic signals. OPG/OCIF strongly inhibited osteoclast formation induced by either 1***α***,25(OH)_2_D_3_, PTH, PGE_2_ or IL-11 in mouse co-culture system.41) Analyses of transgenic mice expressing OPG/OCIF and of animals injected with OPG/OCIF have demonstrated that this factor strongly inhibits osteoclastic bone resorption, resulting in increased bone mass.39),41) In contrast, OPG/OCIF null mutant mice exhibited severe osteopetrosis due to enhanced osteoclastogenesis.42) These results suggested that OPG/OCIF is a physiologically important inhibitor of bone resorption by osteoclasts.41)

## Identification of a long-sought-after ligand, “ODF”

A mouse bone marrow stromal cell line, ST2, is known to support osteoclast formation from mouse spleen cells in the presence of 1***α***,25(OH)_2_D_3_ and dexamethasone (Dex).43) OPG bound to a single class of high-affinity binding sites induced by 1***α***,25(OH)_2_D_3_ and Dex on ST2 cells.41) When the binding sites on the treated ST2 cells were occupied by OPG, the cells failed to support osteoclast formation from spleen cells. These results strongly suggested that the sites are involved in cell-to-cell signalings between stromal cells and osteoclast progenitors, and that OPG inhibits osteoclastogenesis by interrupting the signaling through its binding sites.

**Fig. 6:**
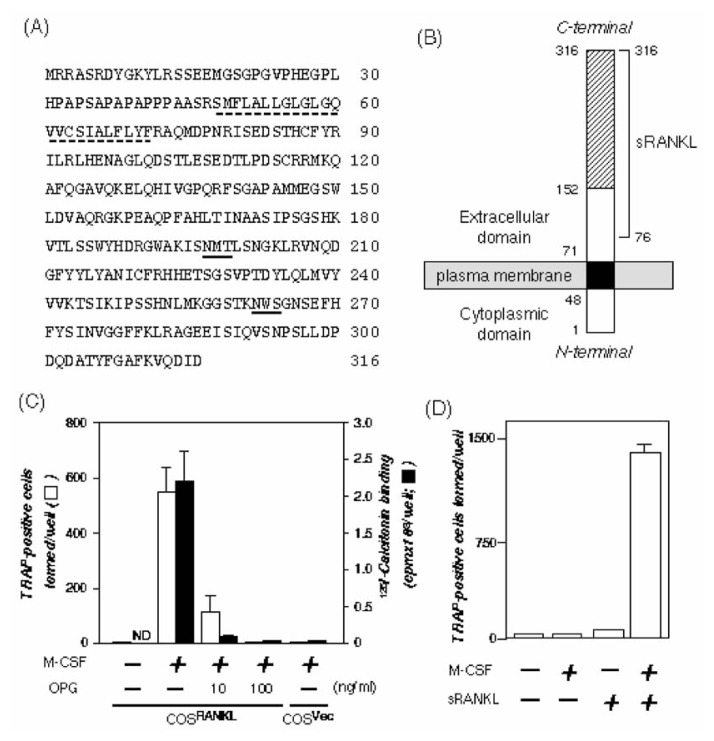
Structure and biological activity of mouse RANKL/ODF. (A) Amino acid sequence of RANKL/ODF. RANKL/ODF consists of 316 amino acid residues. Dotted line indicates the transmembrane domain (TM). Underlines indicate the potential N-glycosylation sites. (B) Schematic structure of RANKL/ODF. RANKL/ODF is a type II transmembrane protein with a short N-terminal cytoplasmic domain (Met^1^-Arg^47^), a single TM (Ser^48^- Phe^71^; closed box), and a longer C-terminal extracellular domain (Arg^72^-Asp^316^). The striped area represents the homologous C-terminal region (Asp^152^-Asp^316^) in the TNF ligand family. sRANKL is prepared by fusing the C-terminal region (Asp^76^-Asp^316^) of RANKL/ODF to the C-terminal end of thioredoxin. (C) Induction of osteoclasts from spleen cells by the fixed RANKL-expressing COS cells. COS-7 cells transfected with the RANKL expression vector (COS^RANKL^) or the empty vector (COS^Vec^) were cultured for 2 days on cover slips in 24-well plates, fixed with paraformaldehyde, and washed with phosphate-buffered saline. Mouse spleen cells (7 × 10^5^ cells) were cultured on the fixed cells in the presence or absence of 10 ng/ml M-CSF and the indicated concentrations of OPG. After culturing for 6 days, the cells were subjected to TRAP staining (open box), and calcitonin binding (closed box). Data are expressed as the means ± SD of six cultures. ND, not determined. (D) sRANKL induces osteoclasts from spleen cells in the presence of M-CSF. Spleen cell were cultured in the presence or absence of M-CSF and/or sRANKL for a week, and then the cells were fixed and stained with TRAP.

Using OPG/OCIF as a probe, a cDNA clone with an open reading frame encoding 316 amino acid residues (Mr 36K) was cloned from an expression library of ST2 cells 44) (Fig. 6A and B). Hydropathy analysis showed the absence of a signal sequence and the presence of an internal 24-residue hydrophobic domain, which presumably represents a transmembrane (TM) domain.44) This structure was typical of a type II transmembrane protein with an extracellular C-terminal region. A homology search of the GenBank sequence database revealed that the C-terminal 165 residues of the protein had a significant homology to the extracellular domains of the TNF ligand family members.44)

Lacey *et al*.45) also cloned the same molecule independently, naming it OPG ligand (OPGL). ODF/OPGL was found to be identical to TNF-related activation-induced cytokine (TRANCE) and receptor activator of NF-***κ*** B ligand (RANKL), both of which were cloned as factors regulating T-cell and dendritic cell functions, respectively.46),47) Thus, ODF, OPGL, TRANCE and RANKL are different names for the same molecule. As a standard nomenclature of the same molecule, the ASBMR President’s Committee on Nomenclature proposed **RANKL**.48)

## RANKL induces differentiation of spleen cells into osteoclasts in the absence of osteoblasts

To examine whether RANKL mediates cell-to-cell signals responsible for osteoclastogenesis, we carried out an *in vitro* osteoclast formation assay by evaluating tartrate-resistant acid phosphatase (TRAP) activity and calcitonin binding, a combination of which is unique to osteoclasts44) (Fig. 6C). When COS-7 cells expressing RANKL (COS^RANKL^) or control COS-7 cells transfected with the empty vector (COS^Vec^) were fixed with paraformaldehyde, and then mouse spleen cells were cultured on those fixed cells for 6 days in the presence of M-CSF, TRAP- and calcitonin receptor-positive cells appeared on the COS^RANKL^ cells, but not on the COS^Vec^ cells. Concurrent addition of OPG to the cultures inhibited the formation of TRAP- and calcitonin receptorpositive cells in a dose-dependent manner.44) These results indicate that RANKL together with M-CSF mediates the cell-to-cell signaling essential for osteoclastogenesis (Fig. 6C).

To further examine the biological effect of RANKL, we produced a genetically engineered soluble RANKL (sRANKL).44) sRANKL together with M-CSF induced osteoclasts from spleen cells alone, and OPG negated the effect of sRANKL (Fig. 6D). Neither osteoblasts/stromal cells nor bone-resorbing factors were required for the osteoclast formation. Furthermore, when these osteoclasts were cultured on dentine slices for 3 days in the presence of sRANKL and M-CSF, numerous resorption pits were formed on the slices. Taken together, these results established that RANKL together with M-CSF mediates an essential signal to osteoclast progenitors for their differentiation into active osteoclasts in the absence of osteoblasts.44),49)

## Molecular mechanisms of osteoclastogenesis

**Fig. 7:**
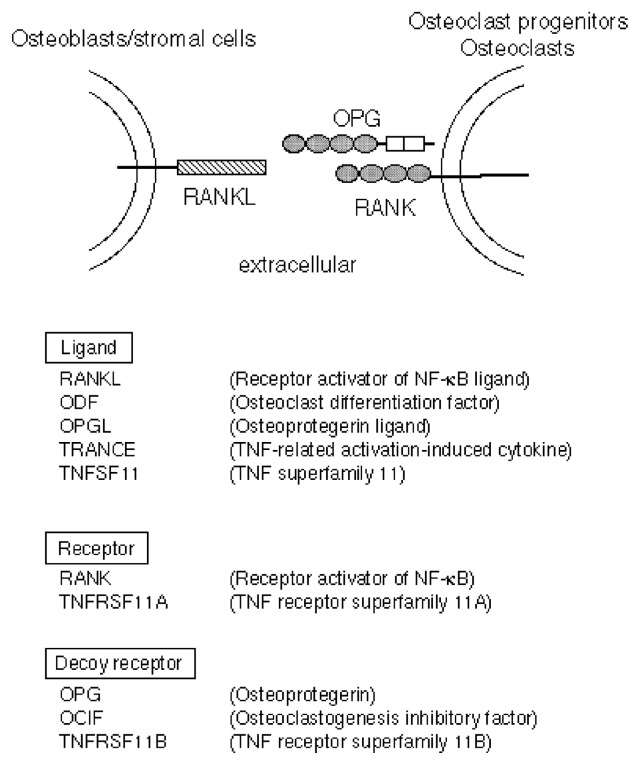
Nomenclature of the ligand, receptor, and decoy receptor of the newly discovered TNF ligand/receptor family members.

The nomenclature of the ligand, receptor, and decoy receptor of the newly discovered TNF ligand/receptor family members has been shown in Fig. 7 according to the recommendation of the ASBMR President’s Committee on Nomenclature.48) RANKL,47) also called ODF,44) OPGL,45) and TRANCE,46) is a new member of the membrane-bound TNF ligand family (TNFSF11), and it is important for osteoclast development as well as lymphocyte development. RANK,47) a new member of the membrane-bound TNF receptor family (TNFRSF11A), has been cloned as a receptor for RANKL in immune systems, and it is the signaling receptor for RANKL in osteoclastogenesis as well.50)–52) OPG/OCIF is a new member of the soluble type belonging to the TNF receptor family (TNFRSF11B), and it functions as a decoy receptor for RANKL.

**Fig. 8:**
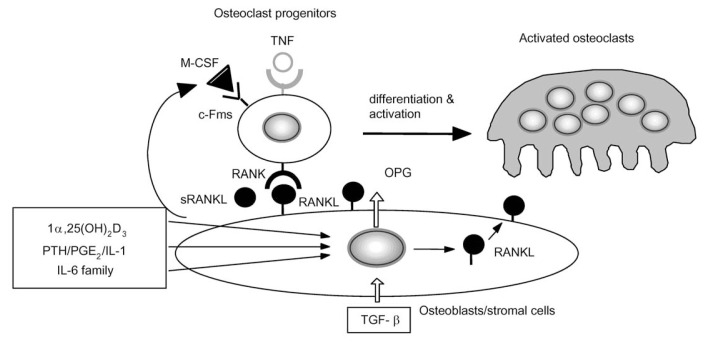
A model illustrating a mechanism by which osteoblasts/stromal cells regulate osteoclast differentiation and activation. Three distinct signals stimulated by 1***α***,25(OH)_2_D_3_, PGE_2_/PTH/IL-1, and the IL-6 family induce RANKL/ODF expression on osteoblasts/stromal cells. RANKL/ODF mediates a signal for osteoclastogenesis through ODF receptor (RANK) expressed on osteoclast progenitors. OPG/OCIF inhibits osteoclastogenesis by interrupting the binding of RANKL/ODF and ODF receptor (RANK). M-CSF produced by osteoblasts/stromal cells is also indispensable for proliferation and differentiation of osteoclast progenitors. Transforming growth factor ***β*** (TGF***β***) inhibits osteoclastogenesis through induction of OPG expression by bone marrow stromal cells.53)

Figure 8 summarizes the molecular mechanisms of osteoclast formation and activation.44),49) Osteoblasts/stromal cells play an essential role in osteoclastogenesis through the expression of RANKL on the membrane by various bone-resorbing factors such as 1***α***,25(OH)_2_D_3_, PGE_2_, PTH, and IL-11. RANKL recognizes osteoclast progenitors having RANK by a mechanism involving cell-to-cell contact. M-CSF produced by osteoblasts/stromal cells is also indispensable for the differentiation of osteoclast progenitors. Osteoclast progenitors differentiate into osteoclasts by binding to RANKL on the osteoblasts/stromal cells. When OPG, a decoy receptor for RANKL, blocks the binding of RANKL to RANK, osteoclast progenitors having RANK are unable to bind to RANKL, thus osteoclast formation is inhibited.

The molecular mechanism of osteoclast formation and activation proposed by *in vitro* studies (Fig. 8) was confirmed by a number of *in vivo* studies including administration of recombinant proteins such as OPG and sRANKL and generation of transgenic (TG) and KO mice of the genes related to the RANKL-RANK signaling (Table I). In addition, loss of function mutations of the OPG gene and gain of function mutations of the RANK gene were reported in patients with skeletal abnormalities, suggesting that the mechanism is applicable to humans as well.

## Clinical trials

The phase I studies of OPG, an Fc-OPG construct (constant fragment of IgG fused to human OPG), suggested that a single subcutaneous dose of OPG was effective in decreasing the levels of bone turnover markers for several days in healthy post-menopausal women70) and patients with breast carcinoma-related bone metastases or multiple myeloma.71) Recently, Bekker *et al*.72) reported that single administration of a fully human monoclonal antibody against RANKL (AMG 162) to osteoporotic patients rapidly and profoundly suppressed bone resorption in post-menopausal women. These results strongly suggest that inhibitors of RANKL-RANK signaling such as OPG and anti-RANKL antibody are useful and applicable to the treatment of metabolic bone diseases such as osteoporosis and rheumatoid arthritis (RA), and other metastatic bone diseases.

At present, anti-TNF therapy using recombinant soluble receptors (Etanercept) and monoclonal antibody (Infliximab) to TNF is available for clinical use, and it works for about two out of three adults with rheumatoid arthritis (RA).73) This therapy reduces the inflammation associated with RA, and may inhibit osteoclastogenesis as well, since TNF is capable of inducing osteoclastogenesis by a mechanism independent of the RANKL-RANK interaction74),75) and also in concert with RANKL.76)

## The biological significance of osteoclastic bone resorption by vitamin D

**Fig. 9:**
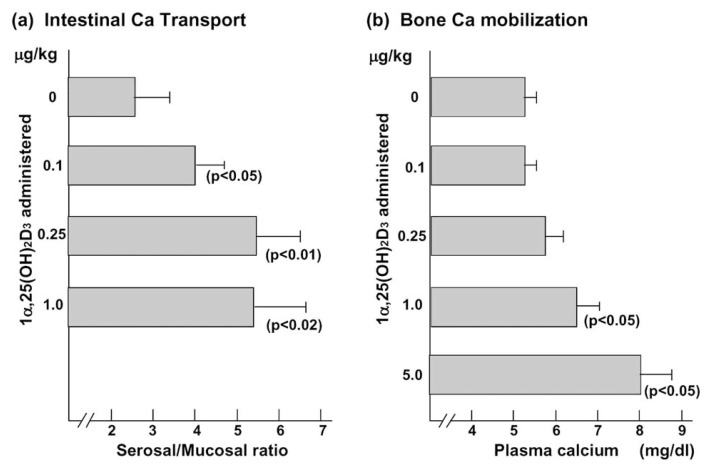
Dose-response effects of 1*α*,25(OH)_2_D_3_ on intestinal calcium transport and bone calcium mobilization activities *in vivo*. Rats were fed a vitamin D-deficient, low calcium diet for 3 weeks, then received graded doses of 1***α***,25(OH)_2_D_3_. Twenty-four hours later, intestinal calcium transport activity (A) and plasma calcium levels (B) were measured.

It is likely that 1***α***,25(OH)_2_D_3_ is a bone-resorbing hormone, but not a bone-forming hormone at least *in vitro*. However, we should emphasize that the *in vivo* bone-mobilizing effects of 1***α***,25(OH)_2_D_3_ depend on its dose levels. Figure 9 shows the differences in the dose levels of 1***α***,25(OH)_2_D_3_ required for inducing intestinal absorption of calcium and bone mineral mobilization activity.77) In this experiment, graded doses of 1***α***,25(OH)_2_D_3_ were administered to rats fed a low calcium, vitamin D-deficient diet. Intestinal absorption of calcium was determined by the routine everted gut sac method, and bone mobilization activity was monitored by measuring serum calcium levels. Intestinal absorption of calcium was stimulated by as little as 0.1 μg/kg body weight of 1***α***,25(OH)_2_D_3_, but bone mobilization activity was induced only by 10–50 times higher doses of 1***α***,25(OH)_2_D_3_. These results indicate that physiological doses of 1***α***,25(OH)_2_D_3_ hardly stimulate bone mobilization. Only pharmacological or toxic doses of 1***α***,25(OH)_2_D_3_ induce bone resorption.77)

The question is the relation between vitamin D and PTH in inducing bone resorption *in vivo*. Of several systemic hormones and local factors affecting bone remodeling, vitamin D and PTH may be the most important factors for regulating bone formation and resorption. In fact, PTH stimulates both bone formation and resorption *in vivo*. Also, pharmacological or toxic doses of 1***α***,25(OH)_2_D_3_ induce bone resorption *in vivo*, but physiological doses of 1***α***,25(OH)_2_D_3_ do not (Fig. 9).

## Conclusions

**Fig. 10:**
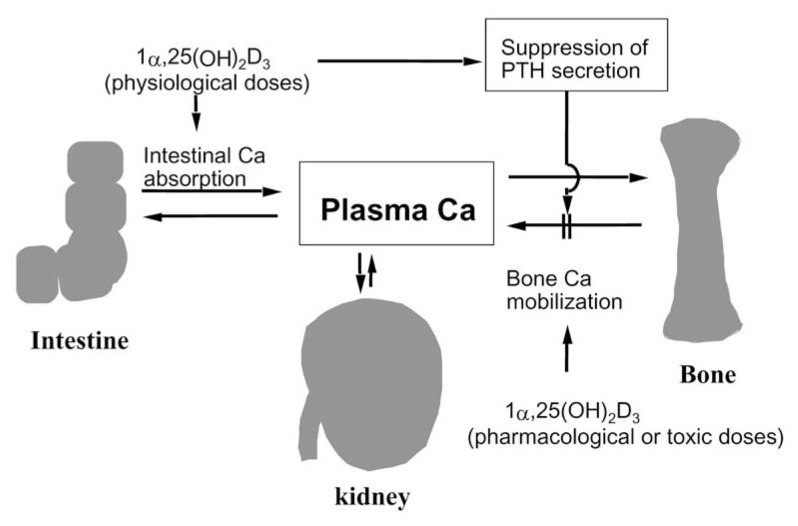
A hypothetical concept of the dose-dependent effects of 1***α***,25(OH)_2_D_3_ on intestinal calcium transport and bone resorption activities *in vivo*.

PTH is essential for generating 1***α***,25(OH)_2_D_3_ in the kidney, which in turn inhibits PTH secretion in the parathyroids (Fig. 10). Thus, it is concluded that vitamin D and PTH working in concert regulate bone formation and resorption to maintain the serum calcium homeostasis.

Physiological doses of 1***α***,25(OH)_2_D_3_ do not stimulate bone resorption *in vivo*. In order for 1***α***,25(OH)_2_D_3_ to induce bone resorption, pharmacological or toxic doses of 1***α***,25(OH)_2_D_3_ are required (Fig. 10). Physiological doses of 1***α***,25(OH)_2_D_3_ preferentially stimulate intestinal absorption of calcium without inducing bone resorption, which then stimulate bone mineralization (Fig. 10). These results support the concept that physiological doses of vitamin D compounds are useful for the treatment of various metabolic bone diseases such as osteoporosis and secondary hyperparathyroidism.

Like other bone-resorbing hormones and cytokines, pharmacological or toxic doses of 1***α***,25(OH)_2_D_3_ stimulate bone resorption. RANKL is required for all the steps of osteoclast development; differentiation, fusion, survival and activation. RANKL mediates signals for osteoclastogenesis through RANK. OPG inhibits the whole processes of osteoclastogenesis as a decoy receptor by interrupting the binding of RANKL to RANK. The discovery of RANKL, OPG and RANK opens a new area of research on bone biology. Further studies on the RANKL-RANK signal transduction pathways will establish new ways for treating several metabolic bone diseases caused by abnormal osteoclast differentiation and function.

## Figures and Tables

**Table I:** Skeletal phenotypes associated with alterations of the RANKL-RANK signaling

RANKL-RANK signaling	Bone resorption			Phenotype
OPG KO42), 54)/mutation (loss of function)55)	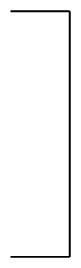	Up	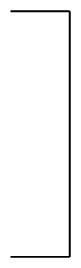	(Paget’s disease) Osteoporosis (FEO, Paget’s disease)
sRANKL administration46), 56)				
sRANKL TG57)				
RANK mutation (gain of function)58), 59)				
OPG TG39)	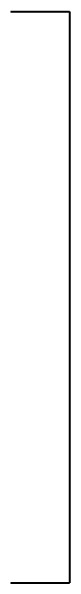	Down	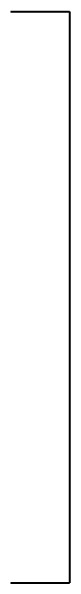	Osteopetrosis
OPG administration39), 41)				
RANKL KO60), 61)				
RANK KO51), 52)				
sRANK TG62)				
TRAF6 KO63), 64)				
c-src KO65)				
c-fos KO66), 67)				
p50/p52 KO68), 69)				
